# Effects of antenatal corticosteroids on fetal hemodynamics: a longitudinal study

**DOI:** 10.1590/0100-3984.2023.0129

**Published:** 2024-05-07

**Authors:** Maria Claudia Bayão Carelli, Fernando Maia Peixoto-Filho, Luis Guillermo Coca Velarde, Renato Augusto Moreira de Sá, Viviane Monteiro, Edward Araujo Júnior

**Affiliations:** 1 Department of Obstetrics, Gynecology, and Fetal Medicine, Perinatal Maternity, Rio de Janeiro, RJ, Brazil; 2 Department of Obstetrics and Fetal Medicine, Instituto Fernandes Figueira (IFF/Fiocruz), Rio de Janeiro, RJ, Brazil; 3 Universidade Federal Fluminense (UFF), Niterói, RJ, Brazil; 4 Department of Obstetrics, Escola Paulista de Medicina da Universidade Federal de São Paulo (EPM-Unifesp), São Paulo, SP, Brazil; 5 Discipline of Human Health, Universidade Municipal de São Caetano do Sul (USCS), Campus Bela Vista, São Paulo, SP, Brazil

**Keywords:** Fetal growth retardation/diagnostic imaging, Adrenal cortex hormones/administration & dosage, Betamethasone/administration & dosage, Ultrasonography, Doppler/methods, Umbilical arteries/diagnostic imaging, Middle cerebral artery/diagnostic imaging, Retardo do crescimento fetal/diagnóstico por imagem, Corticosteroides/administração & dosagem, Ultrassonografia Doppler/métodos, Artérias umbilicais/diagnóstico por imagem, Artéria cerebral média/diagnóstico por imagem

## Abstract

**Objective:**

To study the effect of antenatal corticosteroid administration on fetal hemodynamics using longitudinal analysis of Doppler waveforms in the umbilical artery (UA) and middle cerebral artery (MCA).

**Materials and Methods:**

This was a retrospective study that included 30 fetuses at risk for preterm birth. Twenty-eight pregnant women were treated with betamethasone for fetal lung maturation. Doppler examinations of the UA and MCA were performed once before and three or eight times after corticosteroid administration. We used a Bayesian hierarchical linear model. Reference ranges were constructed, and associations between variables (gestational age and pre-eclampsia) were tested.

**Results:**

The mean maternal age, gestational age at betamethasone administration, and gestational age at delivery were 32.6 ± 5.89 years, 30.2 ± 2.59 weeks, and 32.9 ± 3.42 weeks, respectively. On UA Doppler, there was a significant decrease in the pulsatility index (PI) after corticosteroid administration, with a mean of 0.1147 (credibility interval: 0.03687-0.191) in three observations and a median of 0.1437 (credibility interval: 0.02509-0.2627) in eight observations. However, there was no significant change in the Doppler MCA PI, regardless of gestational age and the presence or absence of pre-eclampsia.

**Conclusion:**

Although antenatal corticosteroid administration induced a significant decrease in the Doppler UA PI, we observed no change in the cerebral vasculature.

## Abstract (pt)

**Objetivo:**

Estudar o efeito da administração antenatal de corticosteroides na hemodinâmica fetal mediante análise longitudinal do Doppler na artéria umbilical (AU) e artéria cerebral média (ACM).

**Materiais e Métodos:**

Este foi um estudo retrospectivo que incluiu 30 fetos com risco de nascimento pré-termo. Vinte e oito gestantes foram tratadas com betametasona para maturação pulmonar fetal. Os exames de Doppler da AU e da ACM foram realizados uma vez antes e depois da administração de corticosteroides, num total de três ou oito observações. Utilizamos o modelo linear hierárquico com abordagem Bayesiana. Foram construídos os intervalos de referência e testadas associações entre variáveis (idade gestacional e pré-eclâmpsia).

**Resultados:**

A média ± desvio-padrão da idade materna, idade gestacional na administração de betametasona e idade gestacional no parto foram 32,6 ± 5,89 anos, 30,2 ± 2,59 semanas e 32,9 ± 3,42 semanas, respectivamente. No Doppler da AU, verificou-se diminuição significativa do índice de pulsatilidade (IP) com a terapêutica com corticosteroides (média: 0,1147 [0,03687-0,191]; em três observações) (mediana: 0,1437 [0,02509-0,2627]; em oito observações). No entanto, não foi observada alteração significativa no IP do Doppler da ACM, independentemente da idade gestacional e do diagnóstico de pré-eclâmpsia.

**Conclusão:**

Os corticosteroides pré-natais induziram diminuição significativa no IP do Doppler da AU, mas não houve alteração na vasculatura cerebral.

## INTRODUCTION

Prematurity is the leading cause of neonatal death and is now the second leading cause of death after pneumonia in children under five years of age. Interventions to reduce death and disability among preterm infants can be applied both during labor and after birth^(1)^. Antenatal corticosteroid treatment (compared with placebo or no treatment) is associated with a reduction in the most serious adverse outcomes associated with prematurity, including perinatal death, neonatal death, and respiratory distress syndrome^(2)^.

A single course of antenatal corticosteroids has been shown to be effective and safe, with no differences in physical and functional development between treated and control survivors up to 30 years of age^(3)^. However, studies have provided preliminary evidence that antenatal steroids administered for the purpose of enhancing fetal lung maturity may induce transient suppression of fetal biophysical activities^(4-7)^. This may seriously compromise the reliability of traditional fetal monitoring techniques^(8)^.

Betamethasone and dexamethasone are potent drugs administered at high doses to pregnant women. There is substantial evidence from animal studies that excessive fetal exposure to glucocorticoids is associated with alterations in fetal cardiovascular and behavioral function, as well as fetal and maternal metabolism and endocrine levels^(9-12)^.

Doppler ultrasonography is a noninvasive technique used in order to assess the hemodynamic components of vascular resistance in pregnancy, especially in those complicated by fetal growth restriction^(13)^.

The aim of this study was to investigate the effect of antenatal glucocorticoid administration on fetal hemodynamics using longitudinal analysis of Doppler waveforms in the umbilical artery (UA) and middle cerebral artery (MCA).

## MATERIALS AND METHODS

This retrospective longitudinal study was conducted over a one-year period (June 2015-May 2016). The study was approved by the local research ethics committee (Reference no. 59296016.0.0000.5269). Because of the retrospective nature of the study, the requirement for informed consent was waived.

Patients were admitted to the semi-intensive unit of a maternity hospital in the city of Rio de Janeiro, Brazil, for fetal and maternal monitoring. All pregnant women received betamethasone for fetal lung maturation and met the following inclusion criteria: carrying a fetus with a gestational age between 24’0 and 33’6 weeks (based on last menstrual period or first trimester ultrasound); and having undergone at least three Doppler measurements, the first one having been performed immediately before corticosteroid administration. Women in whom the fetus had structural, congenital, or chromosomal anomalies were excluded, as were those who did not receive the correct dose of betamethasone (defined as two 12-mg doses given intramuscularly, 24 h apart).

Pregnant women admitted to the semi-intensive unit can have any clinical condition that requires follow-up and in which immediate delivery is not indicated. In such cases, Doppler studies are performed at least twice weekly. However, if there is a clinical comorbidity, these examinations are performed more frequently. All studies were performed with the same device (LOGIQ P6; GE HealthCare, Milwaukee, WI, USA) using a 3.5-MHz convex probe. The pulsatility index (PI) was calculated automatically by the ultrasound machine.

The PIs of the UA and MCA on Doppler studies were analyzed longitudinally with a hierarchical Bayesian model. With this approach, the temporal behavior of fetal hemodynamics and the influence of several variables (gestational age, pre-eclampsia, and gestational age < 32 weeks) were considered, as was the aspect that these variables influence the response at different hierarchical levels.

Two different conditions were considered for the hierarchical model analysis. The first included the pregnant women who underwent three separate post-betamethasone Doppler examinations, and the second included those who underwent eight separate post-betamethasone Doppler examinations. In both models, an initial Doppler examination was performed immediately before corticosteroid administration. Thus, for the first model, we included 30 fetuses for UA Doppler analysis, with 90 post-betamethasone observations, and 27 fetuses for MCA Doppler analysis, with 81 post-betamethasone observations. For the second model, we included 13 fetuses for UA Doppler analysis, with 104 post-betamethasone observations, and 12 fetuses for MCA Doppler analysis, with 96 post-betamethasone observations.

We chose four variables that could affect the PI and denominated their effects as follows: beta 0, referring to the mean change in the PI without the influence of any variable; beta 1, referring to the effects of corticosteroids on the fetus; beta 2, referring to the influence of pre-eclampsia; beta 3, referring to the effects of gestational age; and beta 4, referring to the influence of a gestational age of less than 32 weeks. We recorded the course and outcome of each pregnancy, including the demographic characteristics of the pregnant women, gestational age at betamethasone administration, gestational age at delivery, mode of delivery, birth weight, 5-min Apgar score, and neonatal in-hospital outcome. It should be noted that the statistical analysis model chosen does not include the calculation of *p*-values. A *p*-value quantifies the discrepancy between the data and a null hypothesis of interest, usually the assumption of no difference or no effect. A Bayesian approach allows *p*-values to be calibrated by transforming them into direct measures of the evidence against the null hypothesis, called Bayes factors. Bayes factors represent the relative probability assigned to the observed data under each of the competing hypotheses. Because the Bayes factor is based on the Bayesian approach, which relies solely on the observed sample to provide direct probability statements about the parameters of interest, it is more appropriate for the purpose of hypothesis testing.

Data were transferred to an Excel 2010 spreadsheet (Microsoft Corp., Redmond, WA, USA). Statistical analyses were performed with the open-source software OpenBUGS.

## RESULTS

Maternal, perinatal, and neonatal characteristics of the study population are shown in Table 1. The study included 28 women with 30 fetuses at high risk for preterm birth. Of the 29 infants who were born alive, all had 5-min Apgar scores ≥ 7. There were no neonatal deaths in our study sample.

**Table 1:** Demographic characteristics of 28 pregnant women and 30 fetuses.

Characteristic	Value
Mean maternal age, years, mean ± SD	32.6 ± 5.89
Nulliparous, n (%)	22 (78.6)
Mean gestational age (weeks) at betamethasone administration, mean ± SD	30.2 ± 2.59
Gestational age < 32 weeks, n (%)	18 (60.0)
Mean gestational age (weeks) at delivery, mean ± SD	32.9 ± 3.42
Cesarean section, n (%)	27 (90.0)
Mean birth weight, grams, mean ± SD	1,790 ± 765
Perinatal deaths, n (%)	1 (3.3)
Neonatal intensive care unit admission, n (%)	24 (80.0)
Hypertension in pregnancy, n (%)	14 (46.6)
Preterm rupture of ovular membranes, n (%)	6 (20.0)
Threatened preterm birth, n (%)	6 (20.0)
Intrahepatic cholestasis, n (%)	2 (6.7)
Oligohydramnios, n (%)	2 (6.7)

**Figure 1:**
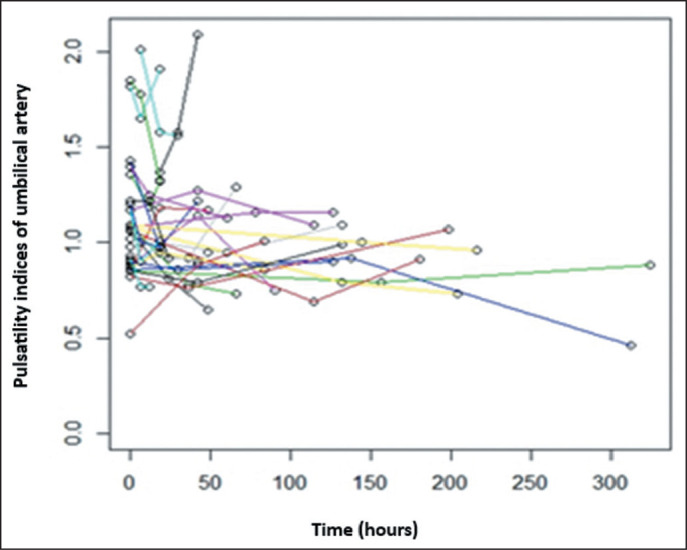
Longitudinal changes in the UA PI in 30 fetuses during serial follow-up after betamethasone administration (three observations per fetus). Each colored line represents an individual fetus.

**Figure 2:**
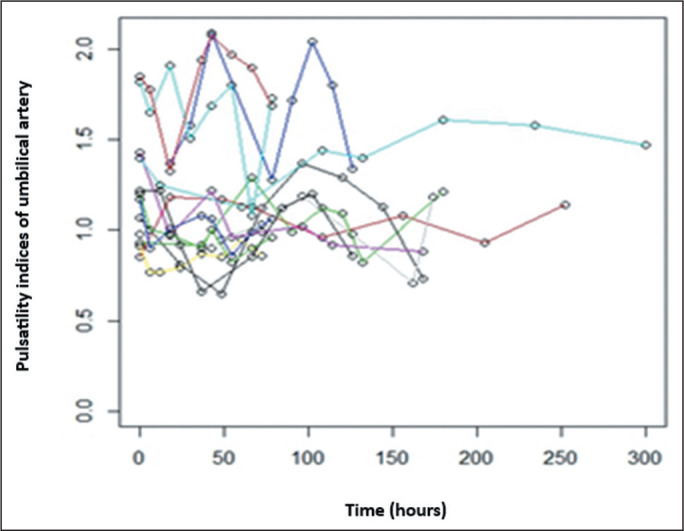
Longitudinal changes in the UA PI in 13 fetuses during serial follow-up after betamethasone administration (eight observations per fetus). Each colored line represents an individual fetus.

**Figure 3:**
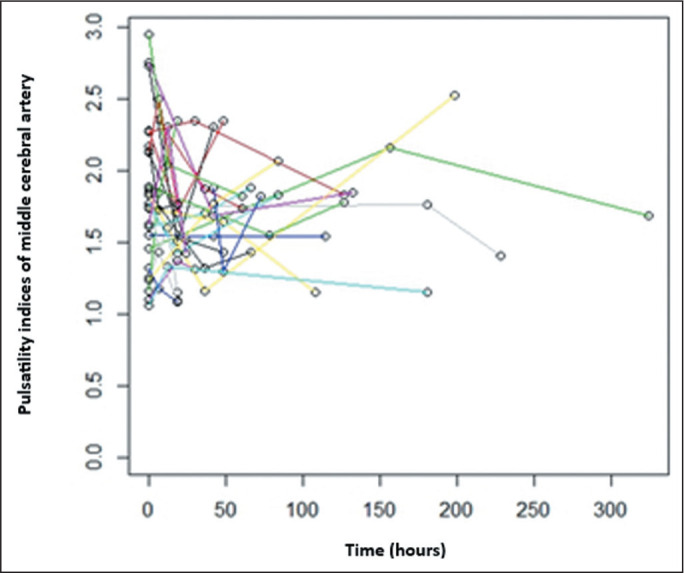
Longitudinal changes in the MCA PI in 27 fetuses during serial follow-up after betamethasone administration (three observations per fetus). Each colored line represents an individual fetus.

**Figure 4:**
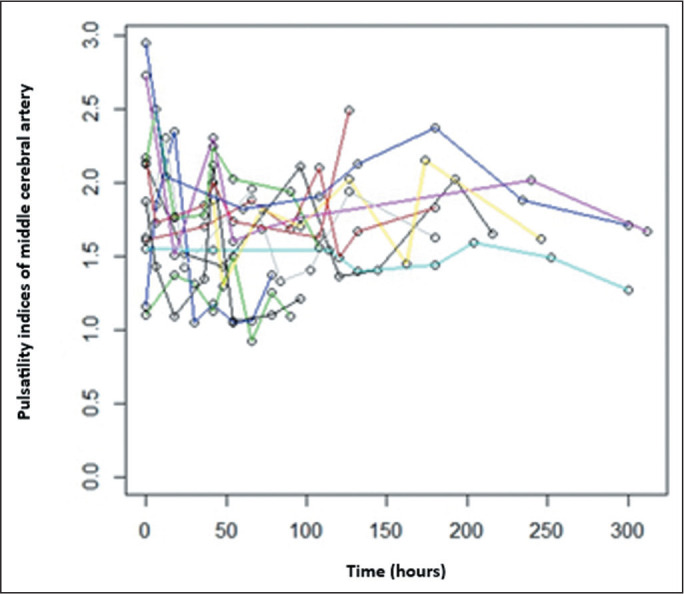
Longitudinal changes in the MCA PI in 12 fetuses during serial follow-up after betamethasone administration (eight observations per fetus). Each colored line represents an individual fetus.

The mean PI for the UA was 0.1147 units higher immediately before corticosteroid administration than in the Doppler examinations performed thereafter (three examinations in this case). In other words, the UA PI was lower after corticosteroid administration (beta 1). That was also observed in the model with eight Doppler examinations. This difference was statistically significant, as can be seen in Tables 2 and 3. There was no null included in the credibility interval (beta 1). Instead, we did not see the same effects for the other variables: pre-eclampsia, gestational age, and gestational age < 32 weeks (beta 2, beta 3, and beta 4, respectively). Figures 1 and 2 illustrate the longitudinal changes in the PI of the UA on Doppler in fetuses during serial follow-up. However, the analysis of the PI of the MCA showed no statistically significant differences after antenatal corticosteroid treatment. As we can see in Tables 4 and 5, the range of percentiles for the variable effects (beta 1, beta 2, beta 3, and beta 4) includes the null. Both models (with three and eight post-betamethasone Doppler examinations, respectively) showed the same results. As we did with the Doppler PI of the UA, we evaluated the longitudinal changes in the Doppler PI of the MCA in fetuses during serial follow-up (Figures 3 and 4).

**Table 2:** Mean UA PI change under the various effects, with credibility intervals, in model 1 (baseline versus three post-betamethasone observations).

Effect	Mean	Median	Credibility interval
Beta 0	-0.4208	-0.4018	[-5.875; 6.906]
Beta 1	0.1147	0.1147	[0.03687; 0.191]
Beta 2	0.405	0.4035	[-0.1623; 0.9843]
Beta 3	0.03477	0.03443	[-0.1874; 0.1986]
Beta 4	0.3455	0.3609	[-0.7428; 1.305]

Beta 0, without the influence of any variable; Beta 1, the effects of the corticosteroid on the fetus; Beta 2, the influence of pre-eclampsia; Beta 3, the effects of gestational age; Beta 4, the influence of a gestational age of less than 32 weeks.

**Table 3:** Mean UA PI change under the various effects, with credibility intervals, in model 2 (baseline versus eight post-betamethasone observations).

Effect	Mean	Median	Credibility interval
Beta 0	1.532	1.728	[-7.561; 10.65]
Beta 1	0.1442	0.1437	[0.02509; 0.2627]
Beta 2	0.3378	0.3455	[-1.208; 1.867]
Beta 3	-0.02758	-0.03286	[-0.3026; 0.2504]
Beta 4	0.1968	0.1949	[-1.792; 2.095]

Beta 0, without the influence of any variable; Beta 1, the effects of the corticosteroid on the fetus; Beta 2, the influence of pre-eclampsia; Beta 3, the effects of gestational age; Beta 4, the influence of a gestational age of less than 32 weeks.

**Table 4:** Mean MCA PI change under the various effects, with credibility intervals, in model 1 (baseline versus three post-betamethasone observations).

Effect	Mean	Median	Credibility interval
Beta 0	-0.5821	-0.5926	[-4.658; 3.161]
Beta 1	0.09208	0.09213	[-0.1706; 0.3537]
Beta 2	0.02946	0.02849	[-0.3585; 0.4209]
Beta 3	0.07138	0.07189	[-0.04237; 0.1942]
Beta 4	0.1951	0.1991	[-0.4047; 0.799]

Beta 0, without the influence of any variable; Beta 1, the effects of the corticosteroid on the fetus; Beta 2, the influence of pre-eclampsia; Beta 3, the effects of gestational age; Beta 4, the influence of a gestational age of less than 32 weeks.

**Table 5:** Mean MCA PI change under the various effects, with credibility intervals, in model 2 (baseline versus eight post-betamethasone observations).

Effect	Mean	Median	Credibility interval
Beta 0	-0.9095	-0.987	[-5.183; 4.048]
Beta 1	0.1439	0.1368	[-0.1668; 0.4937]
Beta 2	-0.2119	-0.2167	[-0.888; 0.5056]
Beta 3	0.09001	0.09203	[-0.06192; 0.2271]
Beta 4	0.2302	0.2505	[-0.7287; 1.028]

Beta 0, without the influence of any variable; Beta 1, the effects of the corticosteroid on the fetus; Beta 2, the influence of pre-eclampsia; Beta 3, the effects of gestational age; Beta 4, the influence of a gestational age of less than 32 weeks.

## DISCUSSION

Betamethasone is a potent drug administered in high doses to pregnant women. Because it is not bound to plasma proteins and is minimally metabolized by the placenta, its concentration in the fetal compartment is relatively high at 2-3 h after treatment^(3)^. The beneficial effects of antenatal steroid administration are greatest when more than 24 h and fewer than seven days elapse between the initial administration of therapy and actual delivery^(14)^. It is estimated that corticosteroids are administered in 70-80% of pregnancies that deliver at 24-34 weeks gestation in lowand middle-income countries^(15)^. Despite the large number of pregnancies exposed to corticosteroids, the short-term effects of maternal steroid administration on fetal cardiovascular status are still relatively unknown^(16)^.

We evaluated the effect of antenatal corticosteroids on UA and MCA indices by Doppler examination. The main finding of our study is that betamethasone therapy has significant effects on the UA PI and no apparent effect on the MCA PI. As in our study, other authors have found a reduction in UA Doppler indices after steroid administration. However, those studies included only pregnancies complicated by fetal growth restriction or absent end-diastolic flow in the UA. In studies showing an improvement in the UA PI after steroid administration, the improvement was transient, lasting 48 h or less^(17-21)^. These findings should be interpreted with great caution by the clinician, especially in fetuses that are hemodynamically critical (absent or reversed end-diastolic flow in the UA Doppler), because they signal an improvement in fetal hemodynamics. The reduction in UA resistance due to the use of betamethasone may give the false impression that the improvement is sustained and encourage less rigorous monitoring in fetuses with a high potential for morbidity and mortality.

Deren et al.^(22)^ and Cohlen et al.^(23)^ performed studies in healthy preterm fetuses. They found that none of the Doppler indices were affected by steroid administration. Shojaei et al.^(24)^ studied fetuses with growth restriction whose mothers received betamethasone. They found that corticosteroids had similar effects on maternal, placental, and fetal arterial blood flow velocity between those with and without pre-eclampsia. Those authors also showed that pre-eclampsia was not a prognostic factor in pregnancies with fetal growth restriction^(24)^.

Unlike the results we obtained on UA Doppler, we observed no significant variation in the PI of the MCA regardless of gestational age. In contrast, Piazze et al.^(25)^ found that in a group of fetuses at < 32 weeks of gestation, the MCA PI decreased significantly at 48 h after and returned to basal values at 96 h after the last dose of betamethasone. However, they found no difference in serial Doppler measurements in the ≥ 32 weeks group. Because of the value of that report, we decided to include gestational age as a variable, as well as the < 32 weeks of gestation subgroup. As previously stated, we observed no significant changes in the PI of the MCA on Doppler studies during betamethasone treatment.

Studies in sheep have shown that maternal corticosteroid administration increases fetal peripheral and cerebral vascular resistance, resulting in increased fetal systemic arterial blood pressure, which may persist for several days, and decreased cerebral blood flow^(3,10-12)^. The underlying mechanisms responsible for the change in human fetoplacental circulation after antenatal betamethasone administration are unclear. It is possible that blood pressure also increases in the human fetus, which could explain the improved fetoplacental perfusion^(21)^. Another possible mechanism for the observed changes in placental vascular resistance is increased placental secretion of corticotropin-releasing hormone, which is thought to be an important regulator of fetoplacental blood flow. One *in vitro* study showed that placental corticotropin-releasing hormone is a potent vasodilator mediated by nitric oxide^(20)^.

Our study had several limitations. It must be emphasized that our group was heterogeneous and that our data are based on a review of medical records. In addition, the Doppler studies were not performed at the same time intervals. For that reason, we chose a longitudinal data analysis, through which we found a significant change in the fetoplacental circulation. However, with this statistical approach we cannot say how long these changes last or when they appeared. Furthermore, some of our patients were being treated with other drugs when the betamethasone was administered, although we do not believe that would explain the changes observed.

We included twin pregnancies in our study, and some authors have questioned the effect of doses used in single pregnancies versus multiple pregnancies^(26)^. However, no difference has ever been demonstrated. In fact, Gyamfi et al.^(27)^ showed that maternal and cord concentrations of betamethasone at birth were similar between single and multiple pregnancies. Therefore, the biological effect is likely to be the same.

Doppler waveform indices such as the PI must be interpreted with caution because they do not fully reflect the dynamics of fetal blood flow and perfusion in the umbilical cord, placental bed or brain vasculature^(3)^. It is not yet known whether alteration of fetoplacental vascular resistance by maternal betamethasone administration has a beneficial effect on fetal prognosis^(28)^, and it should not be interpreted as a sustained improvement in fetal hemodynamics, given that it lasts only approximately two days and could encourage the clinician to be more lax in monitoring hemodynamically critical cases. Such misinterpretation of the hemodynamic effects of antenatal corticosteroids in the umbilical artery could worsen the perinatal prognosis.

## CONCLUSION

Our data contribute to the understanding of fetal physiological responses to corticosteroids. Therefore, in the near future, clinicians may optimize the evaluation of fetal well-being. The reduction in UA resistance caused by antenatal corticosteroids should be taken into consideration.
